# Increase in surgical fixation of pediatric midshaft clavicle fractures since 2008

**DOI:** 10.1186/s12891-021-04918-x

**Published:** 2022-02-23

**Authors:** Elina Sassi, Juuli Hannonen, Willy Serlo, Juha-Jaakko Sinikumpu

**Affiliations:** 1 Department of Children and Adolescents, Oulu University Hospital, Oulu Childhood Fracture and Sports Injury Study, Medical Research Center Oulu, PEDEGO Research Group, University of Oulu, Oulu, Finland; 2 Department of Children and Adolescents, Oulu University Hospital, POB 23, 90029 OYS, Oulu, Finland

**Keywords:** Clavicle, Fracture, Children and adolescents, Operative treatment, Surgical fixation

## Abstract

**Background:**

Clavicle fractures in children have traditionally been treated non-operatively. In adults, a great increase in operative treatment has been reported. We aimed to analyze the respective trend and potential explanatory factors in children.

**Methods:**

This is a single-institution retrospective study in a subregion in Northern Finland. The ICD-code S42.0 was used to identify the cases in the hospital registry. Altogether, 214 children, aged < 16, with consecutive clavicle fractures were first enrolled in the area during 2008–2019. Hospital journals and radiographs were reviewed. After lateral and medial fractures and patients living outside the area were excluded, final study population was 172. The respective population at risk was extracted by Statistics Finland. Predictive factors and annual rates of operative treatment as adjusted for 100,000 children at risk were determined.

**Results:**

The rate of the surgical treatment of clavicle fractures increased from zero in 2008 to 10.8 in 2019 per 100,000 age-adjusted children (β = 0.864, 95% confidential intervals (CI) 0.4 to 1.4). There was a rise in the rate of surgery from 2.6% (2014–16) to 16.1% (2017–19) (diff. 13.5, 95% CI 1.7 to 23.3%). A displacement > 15 mm and a shortening of > 15 mm were associated with the increased risk of surgery but did not change during the study period. Age > 9 years increased the risk of surgery; the mean age increased from 5.5 years (2008–10) to 8.5 years (2017–19). There was a 3.6-fold increase in sports-related fractures (95% CI 7.4 to 26.4). The severity of the fractures did not change.

**Conclusions:**

There has been an increasing trend in the surgical fixation of pediatric middle shaft clavicle fractures since 2008. The available literature does not support the trend.

## Background

Clavicle fractures in children are common as their incidence has been reported at 29–100 per 100,000 annually [3, 19, 28, 30]. Males are predominating, as is usual in childhood injuries in general. Altogether, 69% of clavicle fractures are located in the middle shaft area [26]. Clavicle fractures are usually caused by a fall on the abducted arm or a direct blow, typically occurring in intensive recreational activities such as ice hockey and football.

There is an established practice to treat children’s clavicle fractures non-operatively [15]. This is supported by the extremely low rates of nonunion or delayed union. Strauss et al. reported only four cases with disturbed bone healing among 537 children with a clavicle fracture [33]. Skin laceration was the dominating indication for open reduction internal fixation (ORIF) surgery in a large body of historical data of 939 children during 1983–2002 [16]. Nevertheless, some concern has arisen regarding the potential harm of clavicle malunion in adolescents; there is increasing understanding that a significantly malunited clavicle may not be as asymptomatic as previously thought [27, 29]. The outcomes of closed treatment may be inferior to what has been reported previously [21]. A shortened clavicle changes the biomechnanics of the shoulder girdle [18] and is associated negatively with the long-term patient-reported outcome in adolescent patients [29]. In adults, there is level-A evidence about the particular long-term sequelae after non-operative treatment [21].

There has been a remarkable change in the treatment of midshaft clavicle fractures in adult patients. Surgical treatment has increased as an alternative to non-operative treatment [14]. This trend is explained by the lower rate of malunion and non-union, better function, and faster return to work [5, 23, 37]. .Virtanen concluded in her dissertation in 2014 that surgical treatment is a good option particularly in patients who require the shortest possible time to preinjury activity [36]. In children, aged mean 13 years, open reduction and internal fixation of clavicle fracture resulted in bone healing in all, while none suffered from permanent complications [22]. In a recent controlled study among adolescents, operative care resulted in better functional outcomes than non-operative care [9]. Vander Have et al. also found that plate and screw fixation resulted in shorter healing time and lower complication rate in adolescents as compared with non-operative treatment [35]. Namdari et al. found, in their small series of 14 children, aged 13, that ORIF surgery of the clavicle resulted in high functional outcome scores [24]. While plate and screw fixation is a more common osteosynthesis, intramedullary nailing of the clavicle is another option [8]; however, it does not provide rotational control and carries the risk of nail erosion or migration [4].

The reasons for the recent changes in clavicle fracture treatment in adults are clear, but the respective treatment in immature bone is still a controversial issue. The trend of surgical care is not widely understood in children despite some existing evidence [34]. We hypothesized that surgical fixation has become more frequent compared to non-operative treatment during the last decade. We also aimed to analyze the potential explanatory factors for this trend.

## Methods

**Fig. 1:**
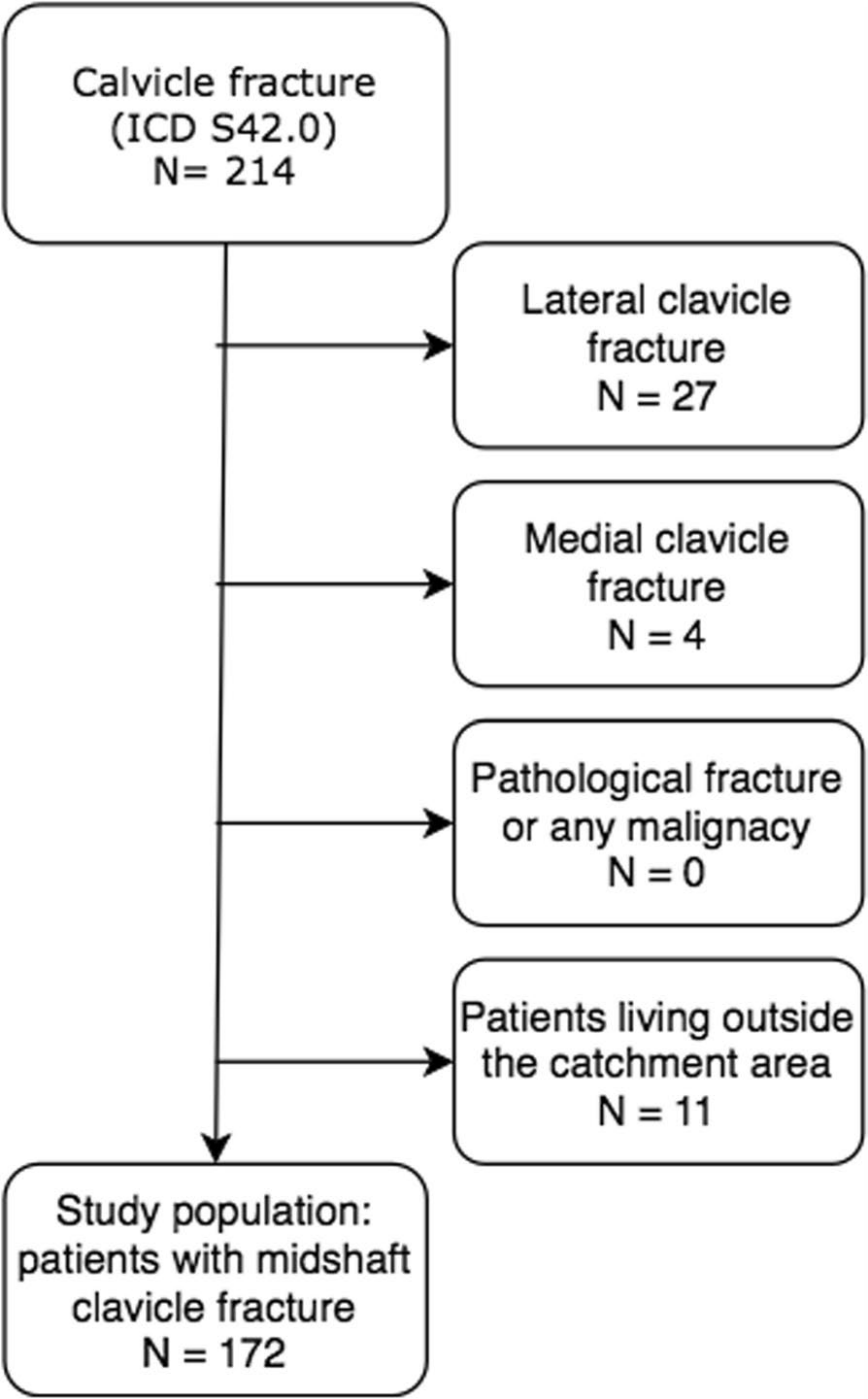
The figure presents a flow chart of the study’s inclusion and exclusion criteria, showing how the study population was selected

The present research is a single-institution study of the surgical fixation of clavicle fractures in children and adolescents, aged < 16 years, in the geographically defined central subregion of the Northern Finland Osthrobothnia Hospital District during 2008–2019. The area comprises nine boroughs in the Oulu surrounding area with a mean respective age-adjusted population at risk of 55.5 (range 52.6 to 57.0). Yearly numbers of the population-at-risk were obtained until 2018, and the population in 2019 was an official assessment by Statistics Finland. Diagnostic code S42.0, according to the International Classification of the Diseases (ICD), version 10, was used to identify the cases in the hospital registry. The primary radiographs of all patients were reviewed to confirm the diagnosis. At first there were 214 patients with a clavicle fracture. Lateral and medial fractures and patients living outside the geographical catchment were excluded. Moreover, pathological fractures and patients with any malignancy were excluded. The primary fractures were counted, while the potential re-fractures during the following 6 months were taken as a complication of the primary fractures. Finally, there were 172 patients comprising the study population (Fig. 1). The study institution is the only round-the-clock pediatric trauma unit in the area, and all patients requiring surgical fixation were assumed to be enrolled.

The incidence of the surgical fixation of midshaft clavicle fractures was reported per 100,000 children annually. The potential change in the incidence of the surgical treatment of clavicle fractures was analyzed yearly using linear regression analysis. The annual proportion of surgically fixed fractures of all clavicle fractures treated in the same institution was also presented as percentages. The patient and injury characteristics of all patients were evaluated. The radiographic findings were recognized, and treatment particulars were reviewed. The highest displacement was measured in millimeters (mm) in a sideways direction and alongside the clavicle axis (shortening). The bone thickness at the fracture site was measured. The angular deformation was measured in degrees between the axes of the medial and lateral fracture fragments. Nearis-software, version 1.10 (Neagen Oy, Finland), was used for viewing and measuring the radiographs. All measurements were primarily made by a radiologist on-duty during the injury; nevertheless, all measurements were reanalyzed by a researcher (ES/JH) for the study purpose. The Inter-observer reliability was excellent, with ICC value 0.993, 95% CI (0.970 to 0.998). The number of follow-up visits and radiographs were evaluated. Bone healing was evaluated by satisfactory callus formation in all visible cortices in the radiographs and fracture line calcification [2]. Unsatisfactory bone healing at three-month and six-month marks were taken as delayed union and nonunion, respectively.

The study cohort was described by using mathematical variables, such as mean, range, and standard deviation (SD). The difference of the distribution of categorical variables (%) between the groups was tested using the chi-squared test or exact test for small groups. The difference in the proportions of independent variables was analyzed using the standardized normal distribution (SND) test. The changes in the risk factors were evaluated by comparing the beginning and the end of the study period using three-year time periods to attain satisfactory groups. The difference in continuous variables between the two groups was tested using an independent-sample t-test. The factors potentially associating with an increased risk (odds ratio [OR]) of operative treatment were analyzed using logistic binary regression analysis. Higher age (> 9 years), higher primary displacement (> 15 mm), greater shortening (> 15 mm), and open fracture (yes/no) were taken as the potential risk factors for surgical fixation.

The 95% CIs were given for all eligible results, and the threshold of statistical significance was taken to be 5% (*p* < 0.05). All *p*-values were two-tailed.

This was a researcher-intended study, and there was no conflict of interest. The patients were not contacted for the study’s purpose, and no ethical board evaluation and approval were thus available. Institutional approval for the study was reached prior to beginning the study. All procedures were performed in accordance with relevant guidelines.

## Results

### The rate of surgical fixation

**Fig. 2:**
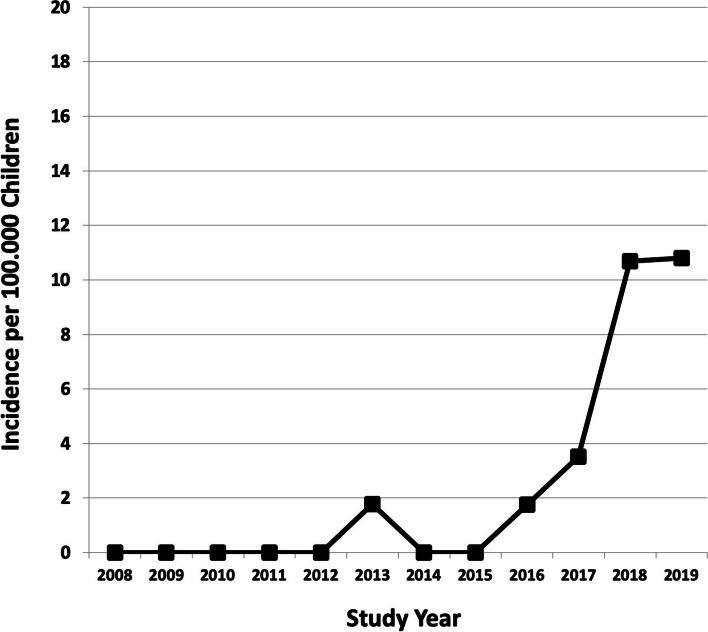
The rate of annual surgical fixation of clavicle midshaft fractures in children aged < 16 years during 2008–2019 as adjusted for 100,000 children at risk in the Northern Finland Hospital District. There has been a jump in surgical care since 2015

The main finding of this study was a steady increase in the incidence of surgical fixation over the 12-year extent of the study period, from zero (2008) to 10.8 (2019) per 100,000 age-adjusted children (β = 0.864, 95% CI 0.4 to 1.4, *P* = 0.004) (Fig. 2). The fractures were exclusively treated by non-operative means from the beginning of the study until year 2013. Thereafter, the incidence of surgical treatment increased from 0.6 per 100,000 children (2014–2016) to 8.3 per 100,000 (2017–2019) (diff. 7.7 95% CI 0.4 to 13.4, *p* = 0.0003). The rate of operative treatment increased from 2.6% of all included cases to 16.1% during the same periods, respectively (diff. 13.5, 95% CI 1.7 to 23.3%, *p* = 0.02).

### Patient and injury characteristics

**Fig. 3:**
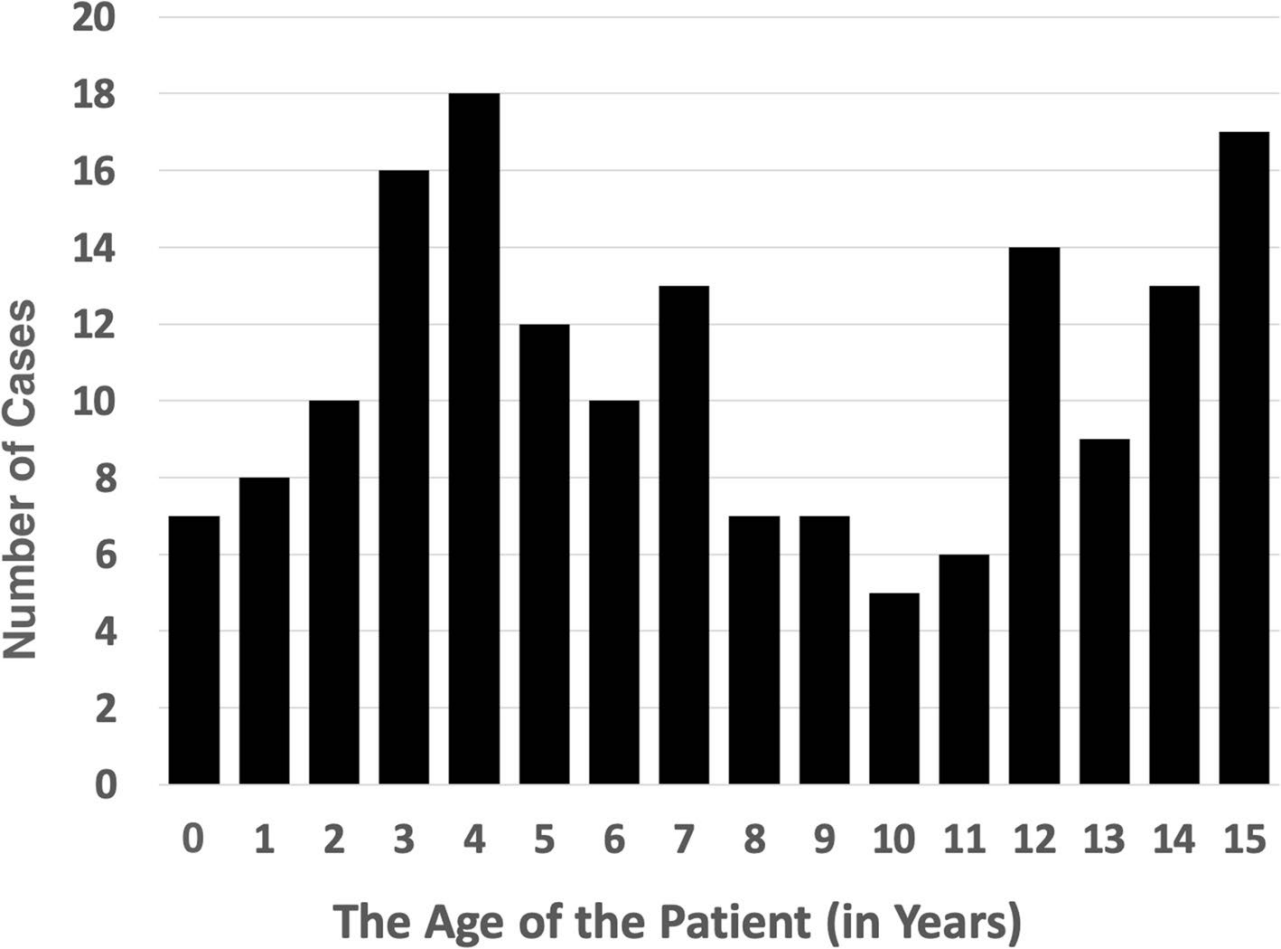
The age distribution of the children (N = 172), < 16 years of age, with clavicle fractures in the study area during 2008–2019

The majority of the patients with midshaft clavicle fracture (67.4%, *N* = 116) were boys. The mean age of the study population was 7.7 years (SD 4.8 years, range 0 to 15) (Fig. 3). In the beginning of the study period (2008–2010), the mean age was 5.5 years, whereas at the end of the study period (2017–2019), the mean age was 8.4 years. The mean age increased 2.9 years during the study period (diff. 2.9, 95% CI 0.9 to 4.9 years, *P* = 0.004) (Table 1). Moreover, most of the fractures occurred on the left side (*N* = 96, 55.8%). The fractures most commonly happened in late autumn (25.6%, *n* = 44, in September–October) or spring (22.0%, *n* = 38, in March–April).

**Table 1:** The comparison of the characteristics of the patients, injuries, fractures and treatment between the beginning of the study period (2008–2010) and the end of the period (2017–2019)

	2008-2010	2017-2019	
	N (%)	N (%)	p-value*
***All patients***	27	87	
***Patient characteristics***			
Age (Mean (SD))	5.5 (4.2)	8.5 (4.7)	**0.01**
Sex			
Male	20 (74.1)	63 (72.4)	0.5
***Injury mechanism***			
Sports injury	2 (7.4)	23 (26.4)	**0.04**
Traffic injury	2 (7.4)	11 (12.6)	0.5
Fall <1m	18 (66.7)	44 (50.6)	0.1
Fall >1m	5 (18.5)	9 (10.3)	0.2
***Fracture characteristics***			
Displacement			
Sideway (mm (SD))	6.9 (2.1)	9.7 (1.1)	0.3
> bone thickness	7 (70.0)	23 (54.8)	0.5
Shortening (Mean (SD))	8.2 (1.7)	13.2 (1.9)	0.2
Shortening > 15mm	0 (0)	8 (27.6)	0.3
Angulation	27.7 (2.7)	27.3 (1.7)	0.9
***Treatment***			
Surgical fixation	0 (0)	14 (16.1)	0.04**
Number of follow-up visits (Mean (SD))	0.8 (0.1)	1.1 (0.1)	0.1
Number of follow-up radiographs (Mean (SD))	1.4 (0.5)	1.7 (1.2)	0.1
Time to free mobilization (weeks) (Mean (SD))	2.4 (0.4)	3.4 (0.6)	0.4

*Tested by Exact test or SND test (**). Student T-test used for continuous variables

All fractures were closed. Local haematoma was visible in 13 cases (7.6%) at the time of the first clinical investigation. Three patients (1.7%) showed primarily slight paresthesia symptoms in the respective upper extremity, but they all recovered spontaneously.

Altogether, 41 injuries (23.8%) were sports related. The proportion of the sports-related fractures increased 3.6-fold during the study period—at the beginning of the study (2008–2010), 7.4% of the fractures were sports related, whereas during 2017–2019, the rate of sports injuries was higher, at 26.4% (*P* = 0.037). Downhill skiing/snowboarding was the most common single recreational activity associated with a fracture (6.4%, *N* = 11). Ten cases (5.8%) had been injured during ice hockey. Four fractures (2.3%) were trampoline injuries and three were based on horse riding (1.7%). Half (*N* = 89, 51.7%) were caused by a conventional fall < 1 m. Traffic injury was the reason for 14.0% (*N* = 24) of the fractures and unspecified fall > 1 m for 18 patients (10.5%) (Table 2).

**Table 2:** Characteristics of the study population

	N	%
***All patients***	172	100
***Sex***		
Male	116	67.4
***Age*** **(Mean, SD) (years)**	7.7	4.8
***Injury side***		
Left	96	55.8
***Injury type***		
Sports injury	41	23.8
Traffic injury	24	14.0
Fall <1m	18	10.5
Fall >1m	89	51.7

### Radiographic findings

Most fractures were nondisplaced, but one in four (26.2%, *N* = 45) were displaced more than the thickness of the bone. The respective bone thickness at the fracture site was mean 9.1 mm (range 4 to 15 mm, SD 2.8 mm). Sideways displacement was mean 9.2 mm (SD 6.6 mm, range 2.0 to 25.0 mm). The average shortening was 11.8 mm (range 2.0 to 35.0, SD 8.6 mm). The mean angular deformity was 28.1° (SD 12.8°, range 6.0° to 70°). There was no change in the severity of the fractures during the study period according to radiographic findings (Table 1).

### Treatment characteristics and associating factors

**Fig. 4:**
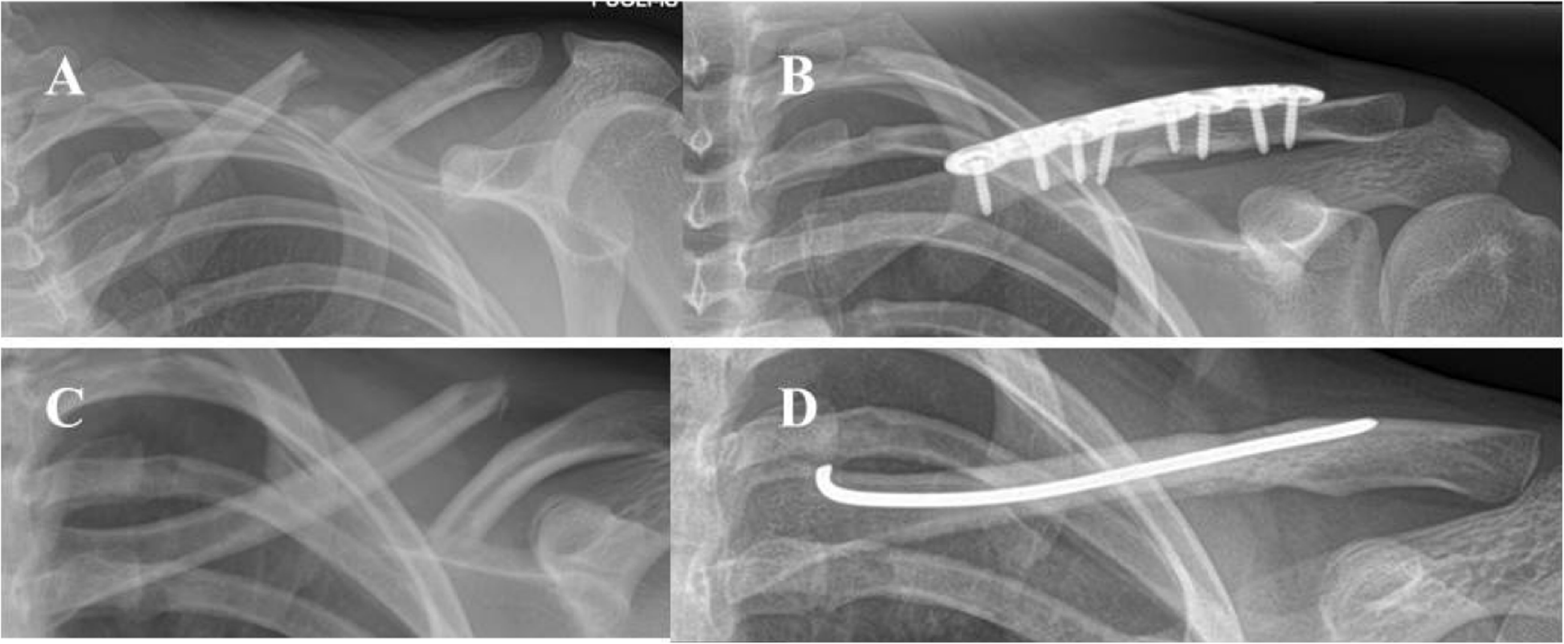
The figure presents the essential surgical methods used in treating middle-third-clavicle fractures by operative means in this study population: Case 1: Preoperative (**A**) and postoperative (**B**) radiographs of a patient operated with plate and screw fixation. Case 2: Preoperative (**C**) radiographs of a patient treated with intramedullary rodding and the respective postoperative radiography (**D**)

Majority of the patients were treated non-operatively. One in 10 of the cases (9.3%, *N* = 16) was treated operatively, eight of them by using plate and screw fixation, and eight by using intramedullary nailing (Fig. 4). Higher age (> 9 years) increased the risk of operation 38-fold (95% CI from 4.9 to 297.8, *P* = 0.001). The mean age of operatively treated patients was 13.6 years, whereas the mean age of the non-operative group was 7.0 years (*P* = 0.003). Greater displacement (> 15 mm) increased the risk of operation 10.5-fold (95% CI of odds ratio from 2.4 to 45, *P* = 0.002) and shortening (> 15 mm) 9.6-fold (95% CI from 2.1 to 43.4, *P* = 0.003). In total, 56.3% (*n* = 9) of operatively treated patients’ fractures were caused by sports injury. In 2011–2013, 14% of the sports-related injuries were treated operatively, whereas the respective rate was 30% in 2017–2019. There was no change in the mean number of follow-up visits per patient during the study time. One patient presented delayed union, and all others healed uneventfully (Table 1).

## Discussion

The main finding of this single-institution retrospective study was that the operative treatment of clavicle fractures became more common in children during the 12 years of study period (2008–2019) (Fig. 2). The children were treated non-operatively at the beginning of the study, but the incidence of surgical treatment had increased to 10.8 per every 100,000 children at risk by the end of the study period. Considering that the incidence of clavicle fractures in children has been reported at 29 to 100 per 100,000, the incidence of surgery (10.8%) is important—meaning that 10.8 to 37% of all pediatric clavicle fractures are operatively treated currently.

Our study confirms that during the last decade (2008–2019), a higher proportion of the patients was treated operatively as opposed to non-operative care. This finding is in steady accordance with the recent reports of adults’ clavicle fractures—the incidence of surgery increased from 1.3 to 10.8 per 100,000 persons in adults from 1987 to 2010 [13]. Huttunen et al. reported a 700% increase in surgical procedures and a 70% increase in the incidence of adults’ clavicle fractures in Sweden over a 10-year period (2001–2012) [14]. Children were not included in their analyses. In Virginia and Boston (USA), adolescent clavicle fractures were increasingly treated with open reduction and internal fixation from 1999 to 2011 [38]. Our findings of an increasing incidence of surgery in pediatric clavicle fractures support not only our hypothesis but also the respective recent literature on adult patients.

Our finding of the increasing incidence of surgical treatment of pediatric clavicle fractures is supported by the fact that adult-type surgical decision making has, in general, slowly but surely been applied to children [22]. Carry et al. asked pediatric orthopedists to evaluate whether the recent adult literature had influenced their clinical decision making in treating clavicle fractures in children. Half of the respondents (48.6%) reported the changed practice in treating their adolescent patients due to recent interest in the clavicle surgery of adults [6]. From this point of view, the increase in the surgical fixation of childhood clavicle fractures during the recent years in this study area was not surprising.

Increased interest in the surgical treatment of clavicle midshaft fractures has been supported by the idea that the results of non-operative treatment may not be as complication-free as previously reported [24]. Clavicle malunion impairs the shoulder girdle, and great shortening may not remodel [1, 11]. The clavicle will remain shorter than on the ipsilateral side, resulting in potential long-term morbidity. It has been suggested that the clavicle achieves most of its length at relatively early childhood, meaning that injuries later in childhood have less remodeling potential [22]. The risk of nonunion may also support surgical fixation albeit the risk is extremely low in children [12]. However, the clavicle’s medial growth plate begins to ossify no earlier than at 18 years and closes at 22–25 years of age [7]. For this reason, the clavicle has remodeling potential up to the mid-20s, which supports conservative treatment despite displacement.

In spite of the traditional preference for non-operative care and very limited or lacking evidence on the superiority of surgical stabilization, there is an increasing number of reports about the positive experience of the surgical stabilization of pediatric clavicles as well. Vander Have et al. reported the positive results of operative treatment in children as compared with non-operative treatment—time to radiographic healing was shorter, and return to the preinjury level of physical activities was faster after operative treatment. Furthermore, five out of 25 who were treated non-operatively suffered from symptomatic malunion, with a mean shortening of 26 mm of the clavicle. In addition, four required secondary corrective operation. The authors stated that plate fixation can restore clavicle anatomy and length, which also supports operative treatment in immature skeletons [35]. Positive results of ORIF surgery in clavicle fractures were reported by Kubiak and Slongo (Bern, Switzerland) as well. Among their 939 patients who presented with a clavicle fracture, 15 children were operatively treated, and all showed bone healing with few minor complications [16]. Namdari reported a series of 14 pediatric patients who were operated for clavicle fracture; all united and showed good recovery with a quick-DASH score of mean 7.0 points [24]. In a single-hospital series of 24 operatively treated clavicle fractures, postoperative satisfaction was 100%, and all united [22]. Hosalkar et al. reported 19 adolescent patients who were all treated by surgical fixation and were satisfied with their decision to undergo surgical care. Full return to sports was achieved in 14 weeks postoperatively (range 12 to 17 weeks) [10]. In contrast, Randsborg et al. evaluated the patient-reported outcomes after the non-operative treatment of 122 patients with a previous clavicle fracture. An Oxford Shoulder score questionnaire, quick-DASH, and VAS overall satisfaction were used. The shortening of the clavicle was associated with a worse Oxford Shoulder score and lower cosmetic and overall satisfaction [29]. During our study period, the portion of a > 15 mm shortening of the fractures increased from 0 to 27.6%. Since shortening is associated with lower overall satisfaction, it may be a possible explanatory factor for the increased rate of surgery, too.

Not only plating but also intramedullary nailing has been reported as a technical option in adolescent clavicle fractures. Frye et al. treated 17 patients with an intramedullary nail, and all fractures showed full bone healing [8]. The rate of major complications after plate fixation has been reported to be low [16, 17]. However, despite increasing reports of good outcomes after clavicle fixation, there is still a lack of level-A research [4]. Furthermore, the published studies of operative treatment in children are based on small groups, and most of them have no control group. Operatively treated children would probably have no or few symptoms if treated non-operatively because of the good prognosis of nonoperative treatment. With all this in mind, it is reasonable that the majority of clinicians treating clavicle fractures in children and adolescents still prefer non-operative care [6, 20]. There is demand for a randomized controlled trial to determine which patients benefit from operative treatment.

We found in this study that there was a 3.6-fold increase in sports-related clavicle shaft fractures from 2008 to 2019. Contact sports such as ice hockey and particular winter sports (alpine skiing and snowboarding) were among the most common recreational activities in the study cases. This is in agreement with the previous understanding. Clavicle fractures in children are known to be associated with high-energy sports injuries: up to half of all operatively treated fractures were sports related among 882 children at Boston Children’s Hospital [34]. In general, children’s participation in organized sports has become more popular in the study country, which can partly explain our finding [25]. This appears reasonable given that active junior athletes and their families are likely to appreciate the shortest possible time to preinjury sports level, and surgical fixation may have therefore been their primary choice.

We also found that the mean age of the patients was higher at the end of the study period. Due to our low numbers of patients, we cannot address this as a fact. The finding of the changed average age needs to be confirmed in another study setting in the future. However, higher age is in line with both the higher need for surgical care and the higher participation in particular sports as it is usually older children and adolescents who are more active in frequent goal-orientated, organized training [25]. It seems that both, a change in recreational activity toward organized sports, such as downhill skiing and ice hockey, and the associated higher age of children, have contributed to an increase in the surgical care of pediatric clavicle fractures. As a conclusion, more preventive interventions should be focused on the coaching of organized junior sports, especially those involving participants > 9–10 years of age.

This is a single-institution retrospective study of surgical care performed in a geographic catchment area with a satisfactorily long study period. As opposed to many epidemiological studies of surgical care that are based on single hospital discharge registries without more detailed information about the individual patients, we reviewed the injury and treatment characteristics of all the enrolled patients. The background factors were available for all, and the patients living outside the catchment area were excluded. Radiographs were available for all, and we were able to exclude with certainty all others than middle-third-shaft fractures. In contrast, epidemiological studies that are based on the ICD code S42.0 from discharge registries cannot distinguish middle-third-shaft fractures from medial or lateral clavicle fractures, and their diagnoses cannot be confirmed. The enrollment of the study material was taken to be inclusive. The respective pediatric population at risk was accurately determined yearly by Statistics Finland. The incidence of surgical treatment was based on the entire children’s population at risk in the area, and the epidemiological findings are reliable. We also determined the rate of operative vs. non-operative treatment yearly, but we are aware that this rate is prone to remarkable change due to the small numbers of cases annually. However, the rate of operatively vs. non-operatively treated cases increased significantly, which is in line with the increase in the incidence of surgical treatment.

The study is subject to some criticism. Despite the relatively large catchment area with approximately 55,500 children, the yearly numbers of operatively treated patients were small in the beginning of the study period. This is reasonable, taking into account the previously unwavering trust in the non-operative treatment of pediatric clavicle fractures [32]. For this reason, the analyses of the characteristics and potential risk factors were performed in three-year periods to attain a satisfactory number of patients per group. We are aware that some isolated cases may have been treated outside the study area, such as during their travels or in private hospitals, but their small numbers would not have affected the conclusion. It is possible that our records are not perfect and that coding errors may have occurred, causing small numbers of cases to be missing. As a limitation, we were not aware of the fundamental reasons why the individual study cases were treated non-operatively or operatively; while we used a retrospective study setting, these primary circumstances were not clear. We agree that the decision making regarding treatment protocol may have been dependent on the preferences of the individual physicians and the operation room resources available that time. Being a single-institution study, these findings cannot be generalized globally. However, this epidemiological approach describes recent authentic changes in the treatment of childhood clavicle fractures in a sufficiently large pediatric population in a setting with a possibility for round-the-clock operational treatment, and our findings align with some earlier epidemiological studies in children and adolescents [34, 38]. A further limitation of the research is the lack of long-term follow-up, which is warranted in future studies as long-term rather than short-term outcomes are more important in evaluating the superiority of any treatment procedure in children’s traumatology [31]. Nonunion, as a short-term complication, is extremely rare, but shortening and malunion may have long-term disadvantageous effects which are not currently widely known. As a limitation, we did not examine our patients postoperatively or have them fill out patient reported outcome measures (PROMs), and therefore we cannot determine if the operative treatment is better than the non-operative. Due to a limited number of operatively treated cases, no subgroup analyses between the different fixation methods were performed in this study.

## Conclusion

This study was important in describing the increase in the incidence of the surgical fixation of pediatric clavicle midshaft fractures over the last decade, which fits well with the recent respective change in the adult population. During the study period, the average age of the patients increased, and their participation in sports increased as well, which both help in explaining the increasing surgery rate. Further level-A studies are warranted to support the increasing trend toward operative treatment even though non-operative treatment is still the gold standard.

## Data Availability

The dataset generated and analyzed during the current study is not publicly available due to a lack of institutional approval for data delivery. However, the dataset is available from the corresponding author on reasonable request.
